# Video-Recorded Airway Suctioning of Clear and Meconium-Stained Amniotic Fluid and Associated Short-Term Outcomes in Moderately and Severely Depressed Preterm and Term Infants

**DOI:** 10.3390/children11010016

**Published:** 2023-12-22

**Authors:** Gazmend Berisha, Line Norman Kvenshagen, Anne Marthe Boldingh, Britt Nakstad, Elin Blakstad, Arild Erland Rønnestad, Anne Lee Solevåg

**Affiliations:** 1 The Department of Paediatric and Adolescent Medicine, Akershus University Hospital, P.O. Box 1000, 1478 Lørenskog, Norway; amboldingh@gmail.com (A.M.B.); blaeli@ous-hf.no (E.B.); 2 Institute of Clinical Medicine, Faculty of Medicine, University of Oslo, P.O. Box 1171, 0318 Oslo, Norway; line.norman.kvenshagen@so-hf.no (L.N.K.); britt.nakstad@medisin.uio.no (B.N.); a.e.ronnestad@medisin.uio.no (A.E.R.); 3 The Department of Anaesthesia and Intensive Care Unit, Stavanger University Hospital, P.O. Box 8100, 4068 Stavanger, Norway; 4 Department of Paediatrics and Adolescent Medicine, Østfold Hospital Trust Kalnes, P.O. Box 300, 1714 Grålum, Norway; 5 Department of Paediatrics and Adolescent Health, University of Botswana, Private Bag, Gaborone 0022, Botswana; 6 Department of Neonatal Intensive Care, Division of Paediatric and Adolescent Medicine, Oslo University Hospital, Rikshospitalet, Nydalen, P.O. Box 4950, 0424 Oslo, Norway; a.l.solevag@medisin.uio.no

**Keywords:** neonatology, newborn life support, depressed infants, suction, short-term outcomes

## Abstract

Background: The aim of this study was to investigate delivery room airway suctioning and associated short-term outcomes in depressed infants. Methods: This is a single-centre prospective observational study of transcribed video recordings of preterm (gestational age, GA < 37 weeks) and term (GA ≥ 37 weeks) infants with a 5 min Apgar score ≤ 7. We analysed the association between airway suctioning, breathing, bradycardia and prolonged resuscitation (≥10 min). For comparison, non-suctioned infants with a 5 min Apgar score ≤ 7 were included. Results: Two hundred suction episodes were performed in 19 premature and 56 term infants. Breathing improved in 1.9% of premature and 72.1% of term infants, and remained unchanged in 84.9% of premature and 27.9% of term infants after suctioning. In our study, 61 (81.3%) preterm and term infants who were admitted to the neonatal intensive care unit experienced bradycardia after airway suctioning. However, the majority of the preterm and more than half of the term infants were bradycardic before the suction procedure was attempted. Among the non-airway suctioned infants (n = 26), 73.1% experienced bradycardia, with 17 non-airway suctioned infants being admitted to the neonatal intensive care unit. There was a need for resuscitation ≥ 10 min in 8 (42.1%) preterm and 32 (57.1%) term infants who underwent airway suctioning, compared to 2 (33.3%) preterm and 19 (95.0%) term infants who did not receive airway suctioning. Conclusions: In the infants that underwent suctioning, breathing improved in most term, but not preterm infants. More non-suctioned term infants needed prolonged resuscitation. Airway suctioning was not directly associated with worsening of breathing, bradycardia, or extended resuscitation needs.

## 1. Introduction

Inability to breathe after birth accompanied by failure to adequately clear the airways of lung liquid is a major cause of neonatal morbidity and mortality [1]. Risk factors of breathing challenges in newborns include preterm birth, intrapartum-related complications, infections, birth defects, and caesarean section delivery (CS) [2,3]. In addition, some maternal diseases and lifestyle factors contribute to a need for resuscitation of the newborn, regardless of delivery mode [4,5].

However, most infants do not require resuscitation at birth [5,6,7]. According to the International Liaison Committee on Resuscitation (ILCOR), the majority of term infants initiate adequate breathing spontaneously or after mild stimulation and drying, while 3% need positive pressure ventilation (PPV). Among the infants in need of PPV, 2% are intubated to further support respiration [8]. The need for resuscitation is higher in preterm infants and dependent on the degree of immaturity [9,10]. Converted to absolute numbers, six million infants need assisted ventilation with a self-inflating bag or T-piece each year [11]. Prolonged, extensive resuscitative action is needed in 1% of these infants [12,13].

The incidence of meconium-stained amniotic fluid (MSAF) worldwide is 10–25% of all births [14,15,16,17], meaning that most infants are born through clear amniotic fluid (CLAF). Routine airway suctioning (ASU) of newly born infants is not recommended, irrespective of whether the amniotic fluid is clear or meconium-stained [18,19,20,21,22,23,24,25,26,27]. The widely referenced observational study [28] that suggests that oro-/nasopharyngeal suction causes vagally induced bradycardia and apnoea is 50 years old. In early 2022, the ILCOR Neonatal Life Support (NLS) Task Force published a systematic review of suctioning of CLAF at birth, and did not confirm that oro-/nasopharyngeal suctioning causes vagally induced bradycardia and/or apnoea [29]. In accordance with this, we have previously shown that suctioning in less depressed infants was not associated with bradycardia, more extensive resuscitation needs, or neonatal intensive care unit (NICU) admission [30].

Participants included in the systematic ILCOR review were predominantly healthy term newborn infants. There is a scarcity of data on depressed infants of different gestational ages (GA) with regard to the potential harmful effects of airway suctioning. In this observational study using transcribed video-recorded neonatal resuscitations, we hypothesised that, in moderately to severely depressed infants, suctioning of CLAF or MSAF negatively affects spontaneous breathing and heart rate (HR) in preterm and term infants, with subsequent need for prolonged resuscitation. Furthermore, we hypothesized that term infants that receive ASU are being admitted to the NICU more frequently than expected, i.e., not due to low birth weight and other predetermined admission criteria. By testing these hypotheses, we hope to contribute to filling the knowledge gaps in our understanding of how delivery room ASU may clinically affect different subgroups of depressed infants.

## 2. Methods

At Akershus University Hospital (AUH), a prospective observational study was performed from August 2014 to November 2016. AUH has a hospital catchment population of 570,000, with yearly deliveries totalling 5000 commencing from 26 weeks of gestation.

### 2.1. The Delivery Ward

All delivery rooms were equipped with a resuscitation bed containing a T-piece and self-inflating resuscitator, a heat convective unit, oxygen mixing device, pulse oximeter, and a suction device powered by gas. Electrocardiogram (ECG) monitoring equipment was not consistently accessible during the data collection period, whereas tools for endotracheal intubation and establishing intravenous access were readily at hand. Suctioning of the airway utilized a suction catheter with a maximum negative pressure of 100 mmHg (13 kPa, 135 cm H_2_O) in accordance with recommendations in [31], while any visible secretions outside of the mouth and on the face were cleared using a towel. Midwives oversaw every phase of labour, excluding anticipated complex deliveries, where one or more obstetricians were in attendance. A paediatric resident was consistently present during the delivery of infants below 35 weeks of gestation and in instances of suspected foetal asphyxia or obstructed delivery. The neonatal resuscitation team included a paediatric resident and a consultant neonatologist (or paediatrician). A NICU nurse was summoned as required. Infants with GA < 35 weeks and/or birth weight < 2000 g were routinely admitted to the NICU.

All staff received instruction in assisted ventilation and adhered to the guidelines for neonatal resuscitation from the Norwegian Resuscitation Council [18], which were derived from the ILCOR, European Resuscitation Council, and Australian and New Zealand Committee on Resuscitation guidelines. Training specifically emphasized updates to the guidelines, particularly those that de-emphasized the use of ASU [30].

### 2.2. Data Collection

Gesture-activated video cameras with audio recording capabilities (Hikvision 2-megapixel IP camera, Hangzhou, China) were installed under the resuscitation bed’s heat convectors. The cameras were set to capture only the infant, not patient monitors or health professionals except for their hands on the infant. The infants taken to the resuscitation beds during the study period, were videotaped and appraised for relevance, and videos with PPV were evaluated after being downloaded to a computer.

### 2.3. Data Processing

A research aide, without knowledge of potential research questions and no practical neonatal resuscitation experience, transcribed the videos using Interact software version 9 (Mangold Int GmbH, Arnstorf, Germany). A paediatrician (AMB) also transcribed twenty recordings. The twenty transcripts were comparable to the research aides transcripts of the same video logs despite no interrater variability test being done.

The transcripts encompassed information on, e.g., PPV, HR as assessed by staff, ASU, spontaneous breathing, chest compression and intubation. Information about the pregnancy, delivery, Apgar scores and NICU admission was retrieved from the patient electronic records (DIPS, Bodø, Norway and CSAM Partus, Oslo, Norway). We were only able to retrieve information about the delivery mode, and no other information, e.g., cardiotocography alterations, foetal distress, or indication for caesarean section due to deficient information about the birth itself. Inclusion criteria in the present study were (1) video-recorded stabilization with corresponding transcript, (2) gestational age 22–42 weeks, (3) Apgar score < 7 at 5 min, and (4) episodes of airway suctioning during resuscitation and stabilization. Apgar scores were allocated by the health care personnel (a paediatrician/neonatologist and/or midwife) present at the resuscitation, and retrospectively documented in the patient electronic record. Suctioning of the nose and mouth was defined as superficial, while deep suctioning was defined as directly in the trachea and/or in an endotracheal tube. Bradycardia was defined as HR < 100 bpm, transient bradycardia as lasting < 2 min (min), and prolonged bradycardia (PB) as lasting ≥ 2 min.

This paper reports data from some transcripts previously reported in [30,32,33]. The primary purpose of the assembled data was to evaluate the effect of “high-frequency, short-span” simulations with facilitator-led debriefings where the video logs were used for learning and quality improvement. ALS, a consultant neonatologist, and GB (first author) scrutinized the transcripts quantitatively and qualitatively.

Statistical analyses were executed in SPSS v29 (SPSS Inc., Chicago, IL, USA). Medians with interquartile ranges (IQR) display continuous data, while numbers with percentages display categorical data. A *p*-value < 0.05 was considered significant. A Mann–Whitney U test was used to check for differences in continuous variables, and a Pearson Chi-square test to test for differences in categorical variables between patient subgroups. Analyses were executed for the entire cohort, as well as for preterm (GA < 37 weeks) and term (GA ≥ 37 weeks) infants. Non-airway suctioned (non-ASU) infants were used for comparison.

### 2.4. Ethical Consideration

The hospital privacy legislation authority and the Regional Committee for Medical and Health Research Ethics South East Norway approved the project (reference 97987). Video recording and evaluation were considered to have quality assurance and to be associated with minimal risk. Thus, the hospital review board approved presumed consent from the parents (reference 14-032). Written information about the study and an opt-out form were given to all women contemplating giving birth at AUH. Parents could also opt out verbally and have the video deleted. A study webpage was publicly available. Video logs could also be deleted without inquiry if health professionals refused participation. Information about health professionals’ age, experience, and training was not assembled because the data should not be able to be linked back to individual healthcare workers. A condition for institutional review board acceptance and approval was that all videos were deleted after transcription.

## 3. Results

**Figure 1:**
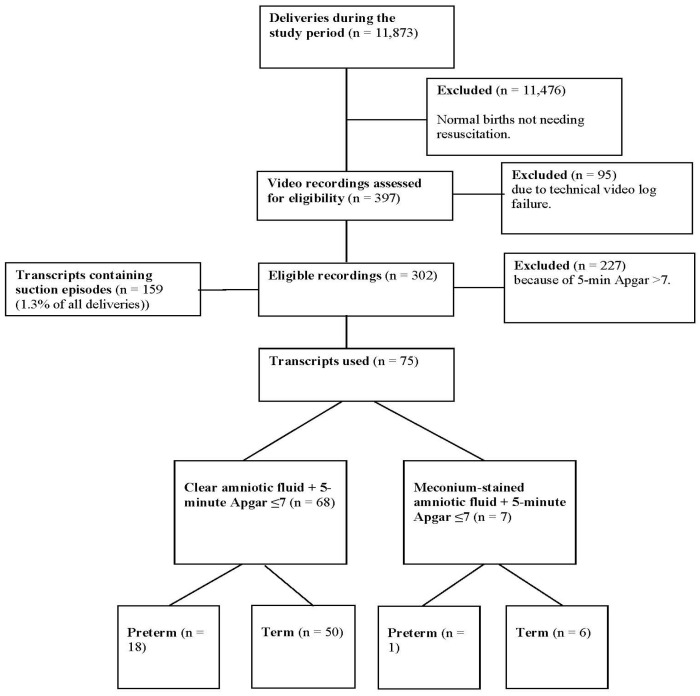
n—number of.

Figure 1 shows the number of deliveries during the study period (n = 11,873), as well as included transcripts in different subgroups. ASU episodes were present in 159 out of 314 transcripts, i.e., 1.3% of all deliveries in the study period. Of the 159 corresponding infants, 75 had a 5 min Apgar score ≤ 7, of whom 68 (90.7%) were delivered through CLAF, and 19 (25.3%) were preterm. Some 16 out of 19 (84.2%) preterm and 18/56 (32.1%) term infants were delivered by CS. For comparison, 26 non-ASU infants with a 5 min Apgar score ≤ 7 were included. In this control group, 3/6 (50.0%) preterm and 9/20 (45.0%) term infants were born by CS. The characteristics of the ASU and non-ASU infants are presented in Table 1.

Two hundred individual suction episodes with a median (IQR) duration of 12 (7–22) sec were identified. The median (IQR) number of ASU episodes per infant was 1 (1–3) and 2 (1–3) in preterm and term infants, respectively, ranging up to 12 episodes in one preterm infant (GA 28 weeks) (Table 1). The preterm infants experienced longer duration of suctioning compared to the term infants, with a median (IQR) duration of 30 (8–47) versus 16 (9–17) sec, respectively. Deep suctioning was performed on 16 (30.2%) vs. 28 (19.0%) of the preterm and term infants, respectively (Table 1).

### 3.1. Effect of ASU on Spontaneous Breathing (Table 2)

In the ASU infants, apnoea was present in 11 (58.0%) of the preterm infants, and 8 (42.0%) had inadequate breathing at birth. In the term ASU infants, 32 (57.1%) had apnoea, while 21 (37.5%) had inadequate breathing at birth. Three (5.4%) term infants had adequate breathing at birth.

Breathing improved from apnoea to inadequate breathing after 107/200 (53.5%) suction episodes, whereas breathing worsened after 7/200 (3.5%) suction episodes, and remained unchanged after 86/200 (43.0%) suction episodes.

Preterm and term ASU infants achieved adequate respiration after median (IQR) 9 (6–13) and 12 (7–16) min, respectively. Adequate respiration in non-ASU preterm infants was achieved after 8 (7–12) min, and after 13 (10–19) min in term non-ASU infants.

**Table 2:** Observations and outcomes regarding respiration comparing different gestational age groups. The patients are presented both as n = numbers and (%).

Variable	Suctioned Preterm Infants GA < 37 Weeks n = 19	Non-Suctioned Preterm Infants GA < 37 Weeks n = 6	*p*-Value	Suctioned Term Infants GA ≥ 37 Weeks n = 56	Non-Suctioned Term Infants GA ≥ 37 Weeks n = 20	*p*-Value
First Breathing Apnoea * Inadequate * Adequate *	11 (58.0) 8 (42.0) 0 (0.0)	3 (50.0) 3 (50.0) 0 (0.0)	0.73	32 (57.1) 21 (37.5) 3 (5.4)	11 (55.0) 8 (40.0) 1 (5.0)	0.98
Respiration before first suction episode (n = 200) Apnoea Inadequate Adequate	30 (56.6) 18 (34.0) 5 (9.4)	N/A	N/A	137 (93.2) 8 (5.4) 2 (1.4)	N/A	N/A
Respiration after first suction episode (n = 200) Apnoea Inadequate Adequate	36 (67.9) 16 (30.2) 1 (1.9)	N/A	N/A	31 (21.1) 98 (66.6) 18 (12.3)	N/A	N/A
Result of suction on respiration (n = 200) Improvement Worsening Unchanged	0 (0.0) 6 (11.3) 47 (88.7)	N/A	N/A	106 (72.1) 0 (0.0) 41 (27.9)	N/A	N/A
Respiratory support before suction (n = 200) PPV CPAP None	39 (73.6) 10 (18.9) 4 (7.5)	N/A	N/A	120 (81.6) 14 (9.5) 13 (8.9)	N/A	N/A
Respiratory support after suction (n = 200) PPV CPAP None	39 (73.6) 13 (24.5) 1 (1.9)	N/A	N/A	122 (83.0) 14 (9.5) 11 (7.5)	N/A	N/A
Suction before first ventilation	4 (21.0)	N/A	N/A	13 (23.2)	N/A	N/A
Ventilation initiated within first minute of age	16 (84.2)	5 (83.3)	0.96	35 (62.5)	15 (75.0)	0.31
Time to adequate respiration (min)	9 (6–13) **	8 (7–12)	0.08	12 (7–16) ***	13 (10–19)	0.85
Endotracheal intubation	8 (42.1)	0 (0.0)	0.05	10 (17.9)	1 (5.0)	0.16
Result of suction on further respiratory support (n = 200) Improvement Worsening Unchanged	0 (0.0) 3 (5.7) 50 (94.3)	N/A	N/A	0 (0.0) 2 (1.4) 145 (98.6)	N/A	N/A
Time resp. supp. episode performed (min)	5 (2–16)	N/A	N/A	4 (2–9)	N/A	N/A

* *Adequate respiration in preterm infants* is defined as sufficient spontaneous respiration with the need for CPAP respiratory support. *Inadequate respiration* is present when preterm infants must be intubated due to no spontaneous respiration, or when CPAP is not sufficient as respiratory support. *Adequate respiration in term infants* is defined as spontaneous respiration without the need for respiratory support. *Inadequate respiration* is present when term infants need CPAP or other initial respiratory support, and when CPAP respiratory support is unsuccessful. ** Eight infants who did not achieve adequate spontaneous respiration at all and were intubated are not included in the statistical analysis. *** Ten infants who did not achieve adequate spontaneous respiration at all and were intubated are not included in the statistical analysis. CPAP—Continuous positive airway pressure. GA—Gestational age. HR—Heart rate. OR—Operating room. N/A—Not applicable. PPV—Positive pressure ventilation. N—Number of.

### 3.2. Effect of ASU on HR (Table 3)

Transient bradycardia was registered in 4 (21.0%) preterm infants, while 11 (58.0%) had PB. In the term infants, 10 (17.9%) had transient bradycardia, while 36 (64.3%) had PB. Chest compressions were performed in 4 (21.0%) preterm and 13 (23.2%) term infants. HR remained unchanged after 190/200 (95.0%) suction episodes. No bradycardia occurred during resuscitation in 14/75 (18.7%) infants receiving ASU.

Time to achievement of HR > 100 bpm was the same in preterm and term infants, regardless of ASU or non-ASU.

**Table 3:** Heart rate (HR) measurements immediately after birth according to gestational age, and its trajectory according to different measures. The HR is presented as median (interquartile range, IQR).

Variable	Suctioned Preterm Infants GA < 37 Weeks n = 19	Non-Suctioned Preterm Infants GA < 37 Weeks n = 6	*p*-Value	Suctioned Term Infants GA ≥ 37 Weeks n = 56	Non-Suctioned Term Infants GA ≥ 37 Weeks n = 20	*p*-Value
FHR Normal Abnormal Not measured	6 (31.6) 12 (63.1) 1 (5.3)	4 (66.7) 2 (33.3) 0 (0.0)	0.30	35 (62.5) 21 (37.5) 0 (0.0)	10 (50.0) 7 (35.0) 3 (15.0)	0.01
1st HR Absent <60 60–100 >100	1 (5.3) 7 (36.8) 5 (26.3) 6 (31.6)	0 (0.0) 4 (66.6) 1 (16.7) 1 (16.7)	0.62	6 (10.7) 17 (30.4) 16 (28.5) 17 (30.4)	3 (15.0) 5 (25.0) 6 (30.0) 6 (30.0)	0.72
Type of bradycardia (brc; min) No brc Transient brc Prolonged brc	4 (21.0) 4 (21.0) 11 (58.0)	1 (16.7) 2 (33.3) 3 (50.0)	0.83	10 (17.9) 10 (17.9) 36 (64.3)	6 (30.0) 4 (20.0) 10 (50.0)	0.45
Infants admitted to NICU after transient and prolonged brc (n = 61 ASU infants; n = 19 non-ASU infants)	15 (78.9)	5 (83.3)	N/A	46 (82.1)	14 (70.0)	
Time to HR > 100 (min)	3 (1–6)	3 (2–6)	1.00	3 (1–5)	3 (1–5)	0.67
HR before suction episode (n = 200) Absent <60 60–100 >100	2 (3.8) 9 (17.0) 7 (13.2) 35 (66.0)	N/A	N/A	4 (2.7) 20 (13.6) 27 (18.4) 96 (65.3)	N/A	N/A
HR after suction episode (n = 200; Total amount with HR < 100 = 65) Absent <60 60–100 >100	2 (3.8) 10 (18.9) 6 (11.3) 35 (66.0)	N/A	N/A	3 (2.0) 18 (12.2) 26 (17.7) 100 (68.1)	N/A	N/A
Result of suction on HR (n = 200) Improvement Worsening Unchanged	1 (1.9) 1 (1.9) 51 (96.2)	N/A	N/A	8 (5.4) 0 (0.0) 139 (94.6)	N/A	N/A
Chest compression	4 (21.0)	0 (0.0)	0.22	13 (23.2)	5 (25.0)	0.87

GA—Gestational age. FHR—Fetal heart rate. Min—Minutes. HR before suction—first HR before the suction procedure where HR < 60 and 60–100 is defined as bradycardia. HR after suction—first HR after suction procedure where HR < 60 and 60–100 is defined as bradycardia. NICU—Neonatal intensive care unit. ASU—Airway suctioned/suction. Non-ASU—Non-airway suctioned/suction. N—Number of. Min—Minutes. N/A—Not applicable.

### 3.3. Prolonged Resuscitation (Table 1)

Resuscitation ≥ 10 min was observed 8 (42.1%) preterm and 32 (57.1%) term ASU infants. In non-ASU infants, resuscitation ≥ 10 min was observed in 2/6 (33.3%) preterm and 19/20 (95.0%) term infants.

### 3.4. NICU Admission (Table 1)

The majority of preterm and term ASU infants and preterm non-ASU infants were admitted, whereas only half of non-ASU term infants were admitted to the NICU (Table 1). Transient or prolonged bradycardia after airway suctioning was seen in 61/62 (98.4%) of the infants who were admitted to the NICU, and in 19/26 (73.1%) non-ASU infants, with 14 (53.8%) of these infants admitted to the NICU (Table 3).

## 4. Discussion

Routine ASU is not recommended; however, airway repositioning and suctioning should be considered if airway obstruction is suspected [34]. In this observational study, immediate outcomes potentially associated with ASU included changes in spontaneous breathing, HR, need for prolonged resuscitation, and NICU admission. The infants were included regardless of clear or meconium-stained amniotic fluid. To address an ILCOR identified knowledge gap, we included depressed preterm and term infants with a 5 min Apgar score ≤ 7. More than half of both preterm and term infants needed resuscitation ≥ 10 min, and >80% were admitted to the NICU for further treatment and observation.

The initial resuscitation steps in the ILCOR algorithm [35] constitute the assessments of tone, breathing and HR, drying, positioning, and tactile stimulation. There has been a sustained debate regarding clearing of the airways by suctioning. Fawke et al. [29] described no clinical benefit, and some evidence for desaturations resulting from suctioning clear amniotic fluid in vigorous infants. Several studies have described apnoea, bradycardia, and delays in achieving normal saturations in relation to airway suctioning [1,15,17,18,30,36,37]. In our study, spontaneous respiration was unchanged after the majority of ASU episodes in preterm infants and improved after almost three quarters of the ASU episodes in term infants. The impact of the depth of the suctioning on spontaneous respiration and HR is mentioned in the studies of Cavalin et al. [30], Purington et al. [24], and Fawke et al. [19]. In our study, the preterm infants underwent both deeper and longer duration suctioning compared to the term infants. We can only speculate whether these factors had an impact on the need for more resusciative measures in our preterm group, as preterm infants in general may have more extensive resuscitation needs.

Associations between suctioning and vagal stimuli resulting in profound bradycardia have been described by Cordero et al. [28]. Other adverse outcomes might also result from suctioning, including local trauma casusing bleeding, oedema, etc. Unfortunately, our transcipts did not contain information about such iatrogenic side-effects. In the Apgar score, HR is the most objective parameter [38]. An increasing HR ≥100 bpm in an initially bradycardic infant together with chest rise are signs of effective ventilation [31]. In our study, 65/75 (86.7%) preterm and term infants experienced bradycardia after ASU (Table 3; “HR after suction episode”). However, the majority of the preterm and more than half of the term infants were bradycardic before the suction procedure was attempted, in spite of guideline recommendations advising against routine suctioning, with ⅙ of the total ASU infants never experiencing bradycardia. In the non-ASU infants, the majority experienced transient or prolonged bradycardia and were admitted to the NICU, most of them being term infants. Even if bradycardia occurred more frequently in the ASU group, it was not significantly higher compared to the non-ASU group, with *p* = 0.82 in the preterm group and *p* = 0.45 in the term group. However, preterm infants had more bradycardia than term infants. A partial explanation might be the maturation with regard to vagal responses among term infants, and that the term ASU infants underwent shorter and less deep suction episodes compared to the preterm Infants.

Noticeably, in our study, more preterm ASU infants were born by acute CS compared to the preterm non-ASU infants. In term infants, more non-ASU infants were delivered by CS. The cardiopulmonary changes associated with vaginal delivery include increasing transpulmonary pressure and increase in the adrenaline-induced Na+-reabsorption that contributes to the aeration of the lungs. This is not present in the same way in the case of CS. Due to this, infants born by CS might present with a larger volume of liquid in the interstitial tissues and airways [39]. In our study, the proportion of infants that required resuscitation for more than 10 min was associated with the proportion born by CS both in the ASU and non-ASU group. Our analyses suggest that ASU is more needed after CS, although a causal relationship cannot be concluded.

Despite the fact that infants received repetitive ASU in our study, we have not been able to prove that ASU was the action causing a need for extended resuscitation and NICU admission in preterm and term infants. More likely than ASU being the culprit, we speculate whether inadequate ventilation and/or difficulty in achieving an open, non-obstructed airway may result in airway suctioning being performed, with an associated need for prolonged resuscitation. In our cohort, preterm infants delivered by CS also needed prolonged resuscitation. Unfavourable outcomes in a compromised preterm infant during birth may result from PB interfering with oxygen delivery, leading to tissue hypoxia. PB might also serve as an indicator of impaired transition at birth and/or inadequate resuscitation [40]. Additionally, ineffective ventilation during neonatal resuscitation may contribute to PB, and hence prolonged resuscitation.

The single-centre and observational features, and the relatively small number of observations, are limitations of this study. The bias risk is thought to be high in observational studies, and the analyses in this study were performed on transcripts, with the source records being deleted before the analyses were performed. Nonetheless, some of the videos were transcribed twice, resulting in a high similarity between transcripts. Nevertheless, in observational studies, the causal relationship between interventions and outcomes are inherently difficult to prove. ASU was performed based on a subjective decision by the caregivers. Decisions regarding CS were taken by the on-call obstetricians, and the infants delivered by CS were either in distress, or delivered on maternal indication, e.g., preeclampsia. The indications for resuscitation measures including ASU, PPV and endotracheal intubation are described in guidelines, but depend on the observations and interpretations of the clinicians. We acknowledge that a 5 min Apgar score ≤ 7 may reflect a vast number of different conditions including congenital anomaly, prematurity, perinatal infection, effects of drugs given to the mother, ineffective resuscitation, or prolonged hypoxia before birth [41].

## 5. Conclusions

In a Norwegian delivery unit, 75 (47.2%) preterm and term infants with a 5 min Apgar score ≤ 7 had ASU performed mostly due to the presence of CLAF. Among the infants that underwent suctioning, breathing improved in most term, but not preterm infants. More non-suctioned term infants needed resuscitation ≥ 10 min. There were more suction episodes in the infants born by CS, and infants born by CS generally needed prolonged resuscitation. Despite the infants receiving repetitive ASU, we were not able to prove that ASU resulted in extended resuscitation needs and NICU admission in preterm and term infants, as outcomes were similar in 26 non-suctioned preterm and term infants.

## Figures and Tables

**Table 1:** Characteristics of the included newborns and details about the suction episodes among the different groups according to gestational age.

Variable	Suctioned Preterm Infants GA < 37 Weeks (n (%) or Median (IQR)) n = 19	Non-Suctioned Preterm Infants GA < 37 Weeks (n (%) or Median (IQR)) n = 6	*p*-Value	Suctioned Term Infants GA ≥ 37 Weeks (n (%) or Median (IQR)) n = 56	Non-Suctioned Term Infants GA ≥ 37 Weeks (n (%) or Median (IQR)) n = 20	*p*-Value
Weight (grams)	1795 (1100–2165)	1746 (1494–2244)	0.64	3668 (3085–4093)	3510 (3111–4130)	0.90
Apgar 1	3 (2–6)	4 (2–7)	0.68	3 (2–6)	3 (2–5)	0.99
Apgar 5	5 (4–7)	7 (6–7)	0.03	6 (4–7)	7 (4–7)	0.18
Apgar 10	8 (7–9)	7 (7–8)	0.83	8 (7–9)	8 (6–9)	0.31
Delivery mode * Vaginal (spontaneous, forceps or vacuum) Acute Caesarean section Elective Caesarean section	3 (15.8) 16 (84.2) 0 (0.0)	3 (50.0) 2 (33.3) 1 (16.7)	0.07	38 (67.9) 15 (26.8) 3 (5.3)	11 (55.0) 7 (35.0) 2 (10.0)	0.70
Suction CLAF MSAF	18 (94.7) 1 (5.3)	N/A	N/A	50 (89.3) 6 (10.7)	N/A	N/A
Suction duration per episode (s)	30 (8–47)	N/A	N/A	16 (9–17)	N/A	N/A
Suction episodes before 1 min of age	18 (34.0)	N/A	N/A	56 (38.1)	N/A	N/A
Reason for suction before 1 min of age Difficult to assess Suspected obstr. airway—indicated Not indicated	7 (38.8) 1 (5.6) 10 (55.6)	N/A	N/A	2 (3.6) 2 (3.6) 52 (92.8)	N/A	N/A
Suction episodes in 1–5 min of age	35 (66.0)	N/A	N/A	91 (61.9)	N/A	N/A
Reason for suction in 1–5 min of age Difficult to assess Suspected obstr. airway—indicated Not indicated	12 (34.3) 2 (5.7) 21 (60.0)	N/A	N/A	36 (39.6) 6 (6.6) 49 (53.8)	N/A	N/A
Suction depth (n = 200) Superficial Deep Superficial plus deep	9 (17.0) 16 (30.2) 28 (52.8)	N/A	N/A	58 (39.5) 28 (19.0) 61 (41.5)	N/A	N/A
Suction total episodes/infant	1 (1–3)	N/A	N/A	2 (1–3)	N/A	N/A
Time of suction episodes performed (min)	3 (2–12)	N/A	N/A	6 (3–9)	N/A	N/A
Total suction episodes all infants (n = 200)	53 (26.5%)	N/A	N/A	147 (73.5%)	N/A	N/A
Extended resuscitation (need for resuscitation > 10 min)	8 (42.1)	2 (33.3)	0.55	32 (57.1)	19 (95.0)	0.002
Infant after resuscitation Normal (given to mother) Neonatal intensive care unit (n = 62 ASU infants; n = 17 non-ASU infants) Died in DR/OR	1 (5.3) 17 (89.4) 1 (5.3)	0 (0.0) 6 (100.0) 0 (0.0)	0.71	10 (17.9) 45 (80.4) 1 (1.8)	9 (45.0) 11 (55.0) 0 (0.0)	0.05
Therapeutic hypothermia	0 (0.0)	0 (0.0)	N/A	5 (9.0)	0 (0.0)	0.16
Outcome at 24 h Normal Neonatal intensive care unit (n = 54 ASU infants; n = 17 non-ASU infants) Died	1 (5.3) 17 (89.4) 1 (5.3)	0 (0.0) 6 (100.0) 0 (0.0)	0.71	18 (32.1) 37 (66.1) 1 (1.8)	9 (45.0) 11 (55.0) 0 (0.0)	0.51
Dead before hospital discharge	4 (21.0)	0 (0.0)	0.22	1 (1.8)	0 (0.0)	0.54
Death Early (day 0–6 from birth) Late (day 8–27 from birth)	4 (21.0) 0 (0.0)	0 (0.0) 0 (0.0)	0.22	1 (1.8) 1 (1.8)	0 (0.0) 0 (0.0)	0.38

* We were only able to retrieve information about the delivery mode, and no other information, e.g., cardiotocography alterations, foetal distress, or indication for caesarean section due to deficient information about the birth itself. GA—Gestational age. IQR—Interquartile range. CLAF—Clear amniotic fluid. MSAF—Meconium-stained amniotic fluid. N/A—Not applicable. ASU—Airway suctioned/suction. Non-ASU—Non-airway suctioned/suction. DR—Delivery room. OR—Operating room. N—Number of. Min—Minutes.

## Data Availability

The data presented in this study are available from the corresponding author upon reasonable request. The data are not publicly available due to privacy restrictions.
